# The relationship between depressive symptoms and medication adherence among individuals with hypertension: An updated systematic review

**DOI:** 10.1192/j.eurpsy.2025.1255

**Published:** 2025-08-26

**Authors:** T. Stamoulis, E. Laiou, K. Dimou, M. Gouva, M. Kourakos

**Affiliations:** 1 Research Laboratory Psychology of Patients, Families and Health Professionals, Department of Nursing, School of Health Sciences, University of Ioannina, Ioannina, Greece

## Abstract

**Introduction:**

Hypertension, a major global public health challenge, is often associated with mental health conditions, particularly depression. Several studies suggest a bidirectional relationship between depressive symptoms and hypertension, which may negatively affect individuals living with hypertension’s ability to maintain essential self-care practices, including adherence to prescribed medications and necessary lifestyle modifications.

**Objectives:**

This study aims to build upon our previous systematic review (doi: 10.5455/msm.2024.36.65-72) by incorporating new data to further investigate the relationship between depressive symptoms and various aspects of self-care, with a particular focus on medication adherence among individuals diagnosed with hypertension.

**Methods:**

The electronic database CINAHL was added to those of PubMed, Scopus, and PsycInfo, with the search extending from April 2023 to the present. Given the substantial diversity among the studies, a narrative synthesis of the results was conducted.

**Results:**

A total of five new studies were included alongside the existing eighteen, involving 11,733 individuals with hypertension who met our eligibility criteria. Among these studies, ten reported a statistically significant association, highlighting the negative impact of depressive symptoms on medication adherence. The remaining thirteen studies did not confirm this association.

**Conclusions:**

This systematic review reaffirms the diverse landscape of research examining the relationship between depressive symptoms and medication adherence in individuals with hypertension. It is advisable to conduct more robust longitudinal studies to thoroughly explore this relationship.

**Disclosure of Interest:**

None Declared

